# Seasonal differences in food and nutrient intakes among young children and their mothers in rural Burkina Faso

**DOI:** 10.1017/jns.2014.53

**Published:** 2014-11-13

**Authors:** Joanne E. Arsenault, Laetitia Nikiema, Pauline Allemand, Kossiwavi A. Ayassou, Hermann Lanou, Mourad Moursi, Fabiana F De Moura, Yves Martin-Prevel

**Affiliations:** 1 Program in International and Community Nutrition, University of California, Davis, CA, USA; 2 Institut de Recherche en Sciences de la Santé, Département Biomédical et Santé Publique, 03 BP 7192 Ouagadougou 03, Burkina Faso; 3 ‘Nutripass’ Research Unit, IRD (Institut de Recherche pour le Développement), Montpellier, France; 4 HarvestPlus c/o International Food Policy Research Institute, Washington, DC, USA

**Keywords:** Diet, Season, Burkina Faso, Nutrient adequacy, EAR, estimated average requirement, MPA, mean probability of adequacy, NPNL, non-pregnant, non-lactating, PA, probability of adequacy, PH, post-harvest

## Abstract

It is important to understand and account for seasonal variation in food and nutrient intakes when planning interventions to combat micronutrient deficiencies in resource-poor settings. The objective of the present study was to quantify food and nutrient intakes and assess the adequacy of micronutrient intakes among young children and their mothers during the lean and post-harvest (PH) seasons in rural Burkina Faso. We quantified food intakes by 24-h recall in a representative sample of 480 children aged 36–59 months and their mothers in two provinces in Western Burkina Faso. We calculated the probability of adequacy (PA) of usual intakes of ten micronutrients and an overall mean PA (MPA). Seasonal changes in nutrient intakes and PA were assessed by mixed linear regression and non-parametric tests, respectively. Energy intakes did not differ significantly between seasons for women or children, although the women's intakes were slightly higher in the PH season. Most of the micronutrient intakes were significantly higher in the PH season, with the exception of vitamin A which was lower and vitamin B_12_ and Zn which were similar across seasons. MPA increased significantly across seasons, from 0·26 to 0·37 for women and from 0·43 to 0·52 for children. PA of Ca, vitamin C, folate and vitamin B_12_ were very low. Staple grains and vegetables were major sources of micronutrients but intakes were not sufficient to meet nutrient needs for the majority of the subjects. Food-based strategies are needed to increase micronutrient intakes of women and children in Burkina Faso.

Seasonal effects on food intake have been described in resource-poor settings with high rates of malnutrition in various parts of Africa^(^^1^^,^^2^^)^ and South Asia^(^^3^^,^^4^^)^. Seasonality in diet in these settings is primarily due to staple cereal harvest periods and depletion of food stocks between harvests. The work of cultivating crops can make an impact on nutritional status by altering energy expenditure^(^^5^^,^^6^^)^. Seasonal morbidity due to malaria and diarrhoea epidemics during the rainy season can also alter nutritional status by decreasing appetite and increasing nutrient needs. In populations with high reliance on agricultural outputs and few resources for acquiring micronutrient-rich animal-source foods, seasonal shifts can have major impacts on micronutrient intakes.

Understanding seasonal variation in food intakes is critically important for accurately assessing the adequacy of nutrient intakes of populations in order to inform the selection and design of proper strategies to reduce micronutrient deficiencies. Although supplementation can effectively reduce micronutrient deficiencies, programme coverage is often low and other strategies such as fortification, biofortification and other food-based interventions may be more sustainable. The availability of quantitative dietary data before programme implementation allows for more accurate estimation of a programme's potential impacts^(^^7^^)^.

We conducted the present study to quantify food and nutrient intakes of women and young children from rural areas of Burkina Faso during the lean and post-harvest (PH) seasons, and to identify nutrient inadequacies in these vulnerable populations. The information from the present study will be used in preliminary planning for potential sorghum biofortification strategies^(^^8^^)^. Quantitative information on sorghum consumption and nutrient intakes and contributions of foods to nutrient intakes was not previously available in Burkina Faso.

## Experimental methods

### Study design and population

The survey was conducted in two rural provinces of Burkina Faso: the Sourou province, in the north Western Region of the Boucle du Mouhoun; and the Sanguié province, in the Western Region of the Centre Ouest. These regions were selected based on a combination of health, agriculture, living conditions and demographics criteria, which included data on sorghum production, household consumption and prevalence of malnutrition. The same households were surveyed in two rounds in 2010, during July − August (rainy, lean season) and November − December (PH season). In the study communities cultivation of crops occurs during the lean season, primarily sorghum, millet, maize and groundnuts, and, to a lesser extent, rice, cotton and market gardening.

The target population consisted of women and preschool children because of their high micronutrient requirements due to their needs for reproduction and growth, respectively. Children aged 36–59 months were selected because at this age they were consuming family dishes, were unlikely to be breastfed, and were more likely to benefit from biofortification due to greater intake of sorghum than younger children. Mothers were recruited along with their children for convenience. One index child from the target age range was randomly selected from a household. The sampling procedure was a multistage cluster selection process of 240 households in each province (five health areas per province × six clusters per area × eight households per cluster). At the first stage, the health areas were randomly selected proportionally to their population size. Population sizes in health areas and villages were obtained from medical districts' records and were further checked for consistency by comparison of the data available from the National Census of 2006. At the next stage, six clusters (villages) were selected in each health area through the same proportional to size technique. Eligible households were identified in each village through a census of all households. Eligible households were those living permanently in the village and having a mother of a child aged 36–59 months who agreed to participate in the study. Among eligible households, the desirable number of households was then selected for the survey using a random number digits table. Informed consent was obtained from the women and/or the heads of households. The study was approved by the National Ethics Committee of the Ministry of Health of Burkina Faso. The sample size was based on the desire to estimate sorghum consumption with a precision of 10 %, using a CV of 0·60 and type I error of 0·05, accounting for a design effect of 1·5 and a 15 % dropout rate.

### Field data collection procedures

Data collection in each cluster took place over a period of 1–2 weeks. A meeting was held with women in each cluster and informed consent was obtained. During the meeting, the methodology of the dietary recalls was explained, recall appointments were scheduled, and plates and bowls were distributed to allow individuals to get used to eating separately from the common meal. Women were shown how to estimate amounts of food consumed by themselves and by their children. Female field workers visited the study homes to conduct the dietary recalls. During the first round (lean, rainy season), sixteen workers conducted dietary recalls due to a constrained time period to complete dietary recalls before the start of Ramadan. During the second round (PH season), eight workers conducted the dietary recalls, six of whom were also involved in data collection during the first round. Recalls were obtained on both weekdays and weekend days, excluding holidays. A separate team of field workers administered a sociodemographic survey a few days apart, not to put too much burden on respondents on the same day.

Dietary data were obtained by the 24-h recall multiple-pass method^(^^9^^)^. Three of the eight households in each cluster were randomly selected to have two 24-h recalls, at least 2 d apart, in order to allow estimation of the distribution of usual intakes and adequacy of nutrient intakes in the population accounting for effects of within-person variation^(^^10^^)^.

Before the start of data collection, information was obtained in the communities on the types of foods and recipes consumed. A set of twenty-one standard recipes was developed based on the most common staple food consumed by households in the study area. All other recipes identified during the recalls were treated as individual household-level recipes and the raw quantities for all ingredients, including water, as well as the total volume of the mixed dish were estimated by the women with the help of the field worker. Probe lists for types of food and recipe ingredients were developed and a predetermined preferred method of measurement was decided for each food or ingredient (i.e. direct weighing, standard size, calibrated household measures, photographs, prices, units, etc.). A set of actual-size validated photographs was prepared for estimating the portion sizes of the most commonly consumed dishes and sauces^(^^11^^)^. The dietary recall methodology was adapted from a previously validated study with weighed food records conducted in Ouagadougou, Burkina Faso^(^^12^^)^.

### Data analysis

Foods consumed were converted to nutrients using CSdietary software (SERPRO and HarvestPlus, 2009). The food composition database was compiled from a previous study by the Institut de Recherche pour le Développement, which was primarily based on the Malian Food Composition Table^(^^13^^)^ and supplemented with data from the WorldFood Dietary Assessment System^(^^14^^)^ and the United States Department of Agriculture National Nutrient Database for Standard Reference (release 20, 2007)^(^^15^^)^. The database was reviewed and updated including Fe content and DM from analysis of food samples conducted at the Institut de Recherche en Sciences de la Santé^(^^16^^)^. Nutrient retention factors in CSdietary were obtained from the United States Department of Agriculture^(^^17^^)^.

Outliers for energy intake (over- or under-reporters) were identified using an adaptation of the arbitrary threshold method proposed by Willett, who suggests fixed cut-off points of < 2090 or > 14640 kJ/d (< 500 or > 3500 kcal/d) for adult women in industrialised countries^(^^18^^)^. For women, we calculated a theoretical BMR for each woman using the formula by the FAO^(^^19^^)^ and subtracted 6 kg if the woman was pregnant, multiplied the BMR by physical activity levels of 2·2 in the lean season (due to workload of cultivating) and 2·0 in the PH season. We then added 1749 kJ/d (418 kcal/d) for pregnant women and 2092 kJ/d (500 kcal/d) for lactating women based on median values of range of additional needs^(^^19^^)^. The cut-points for outliers were determined by dividing the energy requirement by a factor of 0·7 (for under-reporters) and 5 (for over-reporters), which correspond approximately to the cut-points proposed by Willet for a 30-year-old women of 60 kg with a physical activity level of 1·8. The cut-points, depending on the age, weight and pregnant or lactating status of each woman, ranged from 1682–3498 kJ/d (402−836 kcal/d) for under-reporters and 12016–24974 kJ/d (2872−5969 kcal/d) for over-reporters. For children, the average energy requirement was calculated for each child according to sex, age and weight, using the formula provided by the FAO^(^^19^^)^. To estimate cut-points for outliers, we divided the final average energy requirement by a factor of 0·5 for under-reporters and 4·0 for over-reporters; as there is no fixed cut-point method for children, these factors were calculated using the inter-individual CV of energy intakes of 15·1−18·3 %, according to age^(^^20^^)^. The cut-point values for children ranged from 602–1674 kJ/d (144−400 kcal/d) for under-reporters and 4820–13389 kJ/d (1152−3200 kcal/d) for over-reporters (depending on sex and age). The percentage of women excluded as outliers was 4 % (4 % of lactating and 5 % of non-pregnant, non-lactating (NPNL)). Of children, 8 % were excluded as outliers. We examined the results with and without exclusion of outliers and the overall results were not different.

Usual nutrient intakes and the probability of adequacy (PA) were calculated using the probability approach described by the Institute of Medicine^(^^10^^)^ with STATA syntax developed by the Women's Dietary Diversity Project^(^^21^^)^. Adequacy was assessed using the estimated average requirements (EAR) from the WHO/FAO for all micronutrients except Zn and Fe^(^^22^^)^. For Zn, we used the International Zinc Nutrition Consultative Group EAR for a unrefined, cereal-based diet assuming 25 % bioavailability^(^^23^^)^. For Fe, because EAR cannot be estimated due to skewed requirements for young children and NPNL women, we used tables from the Institute of Medicine^(^^24^^)^ that provide ranges of usual Fe intakes associated with probabilities of adequacy and adapted the Fe values for 5 % bioavailability. For the women, we used specific requirements for age and lactation status. The probability approach takes into account the distribution of nutrient requirements of the population^(^^10^^)^. The sd of requirements was calculated using the CV of requirements and the EAR; the CV were 12·5 % for Zn^(^^23^^)^, 20 % for vitamin A, 15 % for niacin, and 10 % for vitamins C, B_6_ and B_12_, thiamin, riboflavin, niacin and folate^(^^25^^)^. The CV for Ca were 20 % for children and 12·5 % for women^(^^26^^)^, and the CV for Fe were 9·4 and 30 % for pregnant and lactating women, respectively^(^^24^^)^. The PA was calculated for each nutrient, ranging from 0 to 1, and an overall mean PA (MPA) was calculated by averaging the PA across the ten nutrients for each individual.

Changes in mean nutrient intakes from the lean and PH seasons were examined by linear mixed models using SAS version 9.3 (SAS Institute Inc.) that took into account the repetition of measures and the sample design by including a random effect for health area; observations were weighted according to size of the population in each province. The variables for PA of each nutrient were too far from a normal distribution to be transformed due to the large number of zero values; therefore, changes in PA were assessed using the non-parametric Wilcoxon sign-rank test using individuals with values at both time periods. The socio-economic characteristics of the children and women did not differ for the subset with data from both seasons from those with data from only one season. The proportion of women who were pregnant and lactating was 12 and 50 %, respectively, in both seasons; therefore, the analysis of nutrient intakes by season was not adjusted for maternal status. Separate analyses were conducted among lactating and NPNL women. The proportions of women and children consuming a food group from various food groupings were compared by season using χ^2^ tests or Fisher's exact test. To examine food sources of nutrient intakes, we used day 1 intakes of foods at the ingredient level in uncooked form and summed the nutrient intakes across all women (and children separately), and summed the nutrient intakes from each food group, and divided the summed nutrient from each food group by the summed total nutrient.

## Results

Energy intakes did not differ between the lean and PH seasons for women or children (Tables 1 and 2, respectively), although the women's intakes were slightly higher in the PH season. Most of the micronutrient intakes were significantly higher in the PH season, with the exception of vitamin A which was lower and vitamin B_12_ and Zn, which were similar across seasons.

**Table 1.** Nutrient intakes of women and probability of adequacy of micronutrient intakes during the lean and post-harvest seasons in rural Burkina Faso (Medians and 25th–75th percentiles)

		Nutrient intake					Probability of adequacy				
		Lean season (*n* 457)		Post-harvest season (*n* 455)			Lean season (*n* 457)		Post-harvest season (*n* 455)		
	EAR*	Median	25th − 75th percentiles	Median	25th − 75th percentiles	*P*†	Mean	Median	Mean	Median	*P*‡
Energy (kJ)	–	8623	6845 − 10548	8874	7523 − 10527	0·10	–	–	–	–	–
Protein (g)	–	73	57 − 96	87	71 − 114	<0·0001	–	–	–	–	–
Fat (g)	–	30	22 − 44	37	25 − 50	<0·0001	–	–	–	–	–
Ca (mg)	800	372	268 − 508	550	398 − 746	<0·0001	0·05	0	0·17	0	<0·0001
Fe (mg)	23·4–49·9	20·9	16·4 − 27·4	26·2	20·9 − 32·4	<0·0001	0·33	0·25	0·49	0·51	<0·0001
Zn (mg)	7–10	11·4	8·9 − 14·0	11·7	9·7 − 14·1	0·12	0·84	1·00	0·88	1·00	0·01
Vitamin C (mg)	38–58	16	10 − 28	24	11 − 45	<0·0001	0·07	0	0·21	0	<0·0001
Thiamin (mg)	0·9–1·2	0·8	0·6 − 1·0	1	0·8 − 1·3	<0·0001	0·21	0	0·39	0·13	<0·0001
Riboflavin (mg)	0·9–1·3	0·9	0·7 − 1·2	1·1	0·9 − 1·4	<0·0001	0·27	0·01	0·44	0·30	<0·0001
Niacin (mg)	11–14	12	9 − 15	15	11 − 19	<0·0001	0·43	0·31	0·66	0·91	<0·0001
Folate (µg DFE)	320–520	150	99 − 220	276	174 − 374	<0·0001	0·05	0	0·20	0	<0·0001
Vitamin B_12_ (µg)	2·0–2·4	0·2	0 − 1·2	0·2	0 − 0·5	0·31	0·13	0	0·02	0	<0·0001
Vitamin A (µg RE)	270–450	237	157 − 392	190	96 − 332	0·001	0·30	0·05	0·21	0·01	0·0002
Overall	–	–	–	–	–	–	0·26	0·21	0·37	0·35	<0·0001

EAR, estimated average requirement; DFE, dietary folate equivalents; RE, retinol equivalents.

* EAR differ according to lactation or pregnancy status; EAR from the WHO/FAO^(22)^ except Zn^(23)^ and Fe^(24)^.

† Differences between lean and post-harvest season were determined by linear mixed models accounting for repeated measures and survey design using transformed nutrient variables.

‡ Differences between lean and post-harvest season were determined by the Wilcoxon sign-rank test using data from women with intakes from both seasons (*n* 434).

**Table 2.** Nutrient intakes of 36- to 59-month-old children and probability of adequacy of micronutrient intakes during the lean and post-harvest seasons in rural Burkina Faso (Medians and 25th–75th percentiles)

		Nutrient intake					Probability of adequacy				
		Lean season (*n* 425)		Post-harvest season (*n* 448)			Lean season (*n* 425)		Post-harvest season (*n* 448)		
	EAR*	Median	25th − 75th percentiles	Median	25th − 75th percentiles	*P*†	Mean	Median	Mean	Median	*P*‡
Energy (kJ)	–	4962	4096 − 5891	4966	4105 − 5740	0·27	–	–	–	–	–
Protein (g)	–	44	35 − 56	50	40 − 67	<0·0001	–	–	–	–	–
Fat (g)	–	21	14 − 29	25	16 − 37	<0·0001	–	–	–	–	–
Ca (mg)	500 − 800	248	171 − 340	367	251 − 505	<0·0001	0·05	0	0·14	0	<0·0001
Fe (mg)	10·8 − 14·8	12·5	9·6 − 15·8	15·6	12·5 − 19·6	<0·0001	0·51	0·55	0·62	0·65	<0·0001
Zn (mg)	2 − 4	6·5	5·3 − 7·9	6·6	5·4 − 8·0	0·72	0·96	1·00	0·95	1·00	0·24
Vitamin C (mg)	25	11	7 − 18	16	8 − 29	<0·0001	0·10	0	0·31	0	<0·0001
Thiamin (mg)	0·4 − 0·5	0·5	0·4 − 0·6	0·6	0·5 − 0·8	<0·0001	0·58	0·79	0·75	1·00	<0·0001
Riboflavin (mg)	0·4 − 0·5	0·5	0·4 − 0·7	0·6	0·5 − 0·8	<0·0001	0·67	0·96	0·77	1·00	<0·0001
Niacin (mg)	4·6 − 9·2	7	5 − 9	9	7 − 12	<0·0001	0·61	0·86	0·67	0·95	0·02
Folate (µg DFE)	120 − 160	91	63 − 139	175	121 − 236	<0·0001	0·28	0	0·64	0·98	<0·0001
Vitamin B_12_ (µg)	0·7 − 1	0·1	0 − 0·8	0·1	0 − 0·3	0·73	0·25	0	0·07	0	<0·0001
Vitamin A (µg RE)	200	168	103 − 248	132	65 − 234	0·02	0·40	0·21	0·33	0·05	0·02
Overall	–	–	–	–	–	–	0·43	0·46	0·52	0·54	<0·0001

EAR, estimated average requirement; DFE, dietary folate equivalents; RE, retinol equivalents.

* EAR differ according to the age of children; EAR from the WHO/FAO^(22)^ except Zn^(23)^ and Fe^(24)^.

† Differences between lean and post-harvest season were determined by linear mixed models accounting for repeated measures and survey design using transformed nutrient variables.

‡ Differences between lean and post-harvest season were determined by the Wilcoxon sign-rank test using data from children with intakes from both seasons (*n* 401).

The MPA of women's micronutrient intakes, over ten micronutrients, was 0·26 in the lean season, increasing to 0·37 in the PH season (*P* < 0·0001; Table 1). PA of women were less than 0·10 for Ca, vitamin C and folate in the lean season and vitamin B_12_ in the PH season. Women's PA were less than 0·50 for most nutrients, except niacin in the PH season, and Zn in both seasons. All PA increased significantly from the lean to PH season except those for vitamins A and B_12_, which decreased.

Of the women, 56 % were lactating (breast-feeding a younger sibling of the child in the present study) and 12 % of the women were pregnant in the lean season. Micronutrient PA were also compared separately for lactating women and NPNL women by season (online Supplementary Table 1); however, pregnant women were excluded due to relatively small numbers for analysis of seasonal changes but were included in the overall estimates for women in Table 1. Lactating women had lower overall PA in both seasons than NPNL women, primarily due to higher nutrient requirements during lactation since absolute nutrient intakes were generally similar (data not shown); however, the MPA increased significantly over the seasons for lactating and NPNL women.

The MPA of children's micronutrient intakes increased from 0·43 in the lean season to 0·52 in the PH season (*P* < 0·0001; Table 2). PA of children were less than 0·10 for Ca in the lean season and vitamin B_12_ in the PH season, and less than 0·50 for folate and vitamin B_12_ in the lean season, and vitamins A and C in both seasons. Changes in PA from the lean to PH season followed the same pattern for children as for the women.

The PA of micronutrient intakes for children were disaggregated by age groups according to dietary reference intakes, which differ for ages 36–47 months and 48–59 months (online Supplementary Table 2). The MPA of younger children was 0·50 in the lean season and increased to 0·60 in the PH season (*P* < 0·0001), while the MPA of older children was 0·34 initially and increased to 0·46 (*P* = 0·0004). PA of Fe and some of the B vitamins such as thiamin, riboflavin and niacin were much higher in younger children than older children who had higher requirements.

**Fig. 1.**
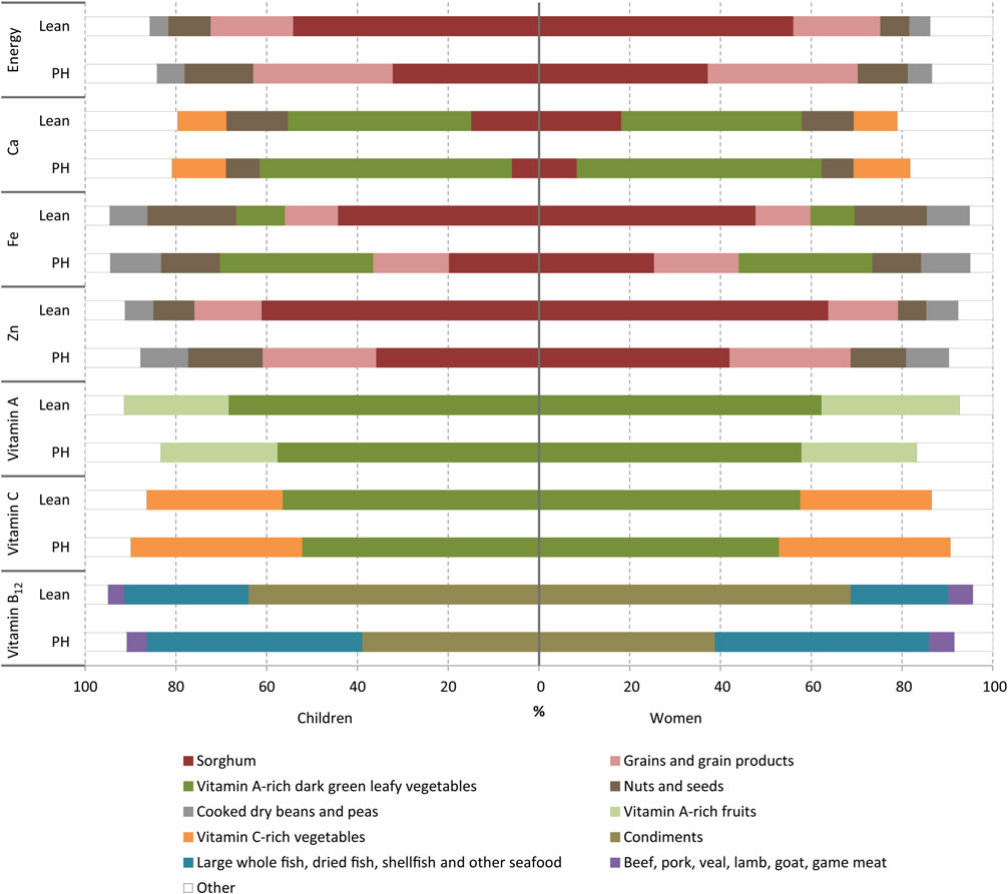
Percentage of selected nutrient intakes contributed by foods or food groups by children and women during the lean and post-harvest (PH) season.

Sorghum was the major source of energy of the diets, particularly in the lean season when it provided 56 and 54 % of energy intakes of women and children, respectively (Fig. 1). Though remaining the major source of energy of the diets, the share from sorghum decreased significantly in the PH season, and the proportion of energy intake from other grains increased from 18–19 % in the lean season to 31–33 % in the PH season. The next highest contributors of energy to the diets were nuts and seeds, providing 6–9 % in the lean season and 11–15 % in the PH season, and beans which provided 4–6 % of energy across seasons. Sorghum and other grains were also the major source of Fe and Zn to the diets in both seasons. Vitamin A was consumed predominately from vitamin A-rich dark green leafy vegetables in both seasons, contributing 62–69 % of vitamin A intakes. The majority of vitamin B_12_ intakes came from fish or condiments that included a fish powder, although the absolute amounts consumed were low as evidenced by the low adequacy of the diets. Consumption of animal-source foods was very low, contributing about 2 % of energy intakes.

## Discussion

The diets of the women and children were low in many important micronutrients across two seasons in rural Burkina Faso. The PA was below 0·50 for many micronutrients. Women had very low PA (< 0·10) for Ca, vitamin C and folate during the lean season, and vitamin B_12_ during the PH season, and children had very low PA for Ca in the lean season and vitamin B_12_ in the PH season. While the PA of most micronutrients increased significantly from the lean to PH season, adequacies still remained relatively low for many micronutrients.

For some micronutrients of major public health importance, adequacy was high (Zn) or moderate (Fe). The majority of Zn and Fe was consumed from sorghum, other staple grains, nuts, seeds and beans that all contain phytate, which interfere with absorption of these nutrients. Although we accounted to some extent for lower bioavailability of Zn and Fe in assessing adequacy, there is not clear international agreement on how to best account for reduced bioavailability, particularly for children^(^^27^^,^^28^^)^.

The diet patterns of children and their mothers were quite similar, with similar proportions of energy and nutrients consumed from grain staples as well as other foods, as the children consumed the same dishes as their mothers. When children start to eat more of the family dishes rather than special infant porridges with one main cereal ingredient, diet diversity increases^(^^29^^)^. In fact, the children (as well as the mothers) had slightly more diverse diets in the PH season than the lean season, with dietary diversity increasing from a mean of 3·64 food groups (out of seven) in the lean season to 3·88 in the PH season. Diet diversity is associated with higher micronutrient intake adequacy in children and women in resource-poor settings^(^^21^^,^^30^^)^.

The overall nutrient adequacy of the women during the PH season was similar to those previously reported for urban Burkinabe women; however, the urban women had higher adequacies of vitamins A and C, and Ca and Zn^(^^31^^)^. The authors note that the urban diet of the women included meat and frequent snacking on mango that was in season at the time. Another study among urban women in Burkina Faso reported an increase in overall micronutrient adequacy from the lean to PH seasons which the authors attributed to the consumption of more vegetables and foods prepared at home^(^^32^^)^.

Food sources of nutrients of children in the present study are similar to those reported by Avallone *et al.*^(^^33^^)^. The main sources of energy in diets of children aged 12–59 months residing in a rural area of Burkina Faso were starchy dishes such as millet-based *tô*^(^^33^^)^. Fe sources were primarily *tô* and sauces made with dried okra and red sorrel leaves, which is in agreement with our data. In another study of younger children (aged 6 months to 3 years) in rural Burkina Faso, vitamin A intakes were slightly higher in the dry than rainy season, seemingly due to the availability of mango^(^^34^^)^. The present study also found that the consumption of dried leaves was a primary source of vitamin A in the rainy season, although the frequency and amounts consumed were insufficient to achieve a high level of adequacy. In this study, intakes of vitamin A were lower in the PH season, mainly because of the higher consumption of dark green leafy vegetables in the rainy season.

The present study was strengthened by the careful design and conduct of the dietary recall methodology due to the prior knowledge of the investigators conducting similar studies in Burkina Faso. The analysis of dietary intakes used the best-practice methodologies of calculating usual intakes and assessment using the probability approach^(^^10^^)^. The present study also benefited from the close supervision of the protocol that contributed to low rates of dropout. Perhaps most importantly, comparison of dietary intakes from two seasons among the same individuals is fairly rare and improves the ability to assess seasonal effects. The present study provides quantitative information about nutrient intakes and adequacy during two seasons and demonstrates the potential bias that would occur if dietary information were captured during only one season.

The present study also had some limitations. Despite our efforts, the 24-h recall method has some limitations. It relies on participant recall of foods consumed and estimation of portion sizes. The conduct of 2 d of recalls may not be sufficient to estimate usual intakes of some nutrients. Dietary recalls also suffer from a need to assess under- and over-reporting, and the exclusion of individuals is debatable, but we chose to use less strict criteria than other methods to limit bias. Our assessment of nutrient intake adequacy is dependent on the established EAR for nutrients, which are not universally agreed upon. We chose to use primarily the WHO/FAO nutrient requirements which are used widely internationally, and while agreeing with Institute of Medicine guidelines for most nutrients do vary for other nutrient such as vitamin A^(^^19^^,^^24^^)^. As noted previously, there is some controversy over dietary Zn requirements^(^^27^^,^^28^^)^. Lastly, the present results are not representative of the entire country because we selected two study areas for the purpose of including areas with a high level of sorghum production.

### Conclusion

In conclusion, grains, nuts and vegetables provide the majority of micronutrients to the diets of women and children in rural Burkina and account for many of the seasonal shifts in micronutrient intake adequacy. Nevertheless, the grain- and vegetable-based diet with few animal-source foods was probably responsible for inadequacies of Ca, Fe and vitamin B_12_ in the women and children. Given the prohibitive cost of animal-source foods to the rural poor, strategies such as biofortification to increase the nutrient content of staple grains and vegetables and to increase bioavailability should be investigated in this setting. In addition, targeted interventions, such as supplementation or food fortification, to improve dietary adequacy during the lean season when agricultural production is limited are warranted.

## Supplementary material

To view supplementary material for this article, please visit http://dx.doi.org/10.1017/jns.2014.53
